# Acetylsalicylic Acid Prevents Intermittent Hypoxia-Induced Vascular Remodeling in a Murine Model of Sleep Apnea

**DOI:** 10.3389/fphys.2018.00600

**Published:** 2018-05-24

**Authors:** Monique C. Suarez-Giron, Anabel Castro-Grattoni, Marta Torres, Ramon Farré, Ferran Barbé, Manuel Sánchez-de-la-Torre, David Gozal, Cesar Picado, Josep M. Montserrat, Isaac Almendros

**Affiliations:** ^1^Laboratori del Son, Servei de Pneumologia, Hospital Clínic, Barcelona, Spain; ^2^Respiratory Department, Hospital University Arnau de Vilanova and Santa Maria, IRB Lleida, University of Lleida, Lleida, Spain; ^3^Centro de Investigación Biomédica en Red de Enfermedades Respiratorias, Madrid, Spain; ^4^Unitat de Biofísica i Bioenginyeria, Facultat de Medicina i Ciències de la Salut, Universitat de Barcelona, Barcelona, Spain; ^5^Institut d'Investigacions Biomèdiques August Pi i Sunyer, Barcelona, Spain; ^6^Section of Pediatric Sleep Medicine, Biological Sciences Division, Department of Pediatrics, Pritzker School of Medicine, The University of Chicago, Chicago, IL, United States; ^7^Department of Pneumology and Respiratory Allergy, Hospital Clinic, Barcelona, Spain

**Keywords:** intermittent hypoxia, sleep apnea, obstructive, acetilsalicilic acid, cardiovascular diseases, aortic remodeling

## Abstract

**Study objectives:** Chronic intermittent hypoxia (CIH), a hallmark feature of obstructive sleep apnea (OSA), induces accelerated atherogenesis as well as aorta vascular remodeling. Although the cyclooxygenase (COX) pathway has been proposed to contribute to the cardiovascular consequences of OSA, the potential benefits of a widely employed COX-inhibitor such (acetylsalicylic acid, ASA) on CIH-induced vascular pathology are unknown. Therefore, we hypothesized that a common non-selective COX inhibitor such as ASA would attenuate the aortic remodeling induced by CIH in mice.

**Methods:** 40 wild-type C57/BL6 male mice were randomly allocated to CIH or normoxic exposures (N) and treated with daily doses of ASA or placebo for 6 weeks. At the end of the experiments, intima-media thickness (IMT), elastin disorganization (ED), elastin fragmentation (EF), length between fragmented fiber endpoints (LFF), aortic wall collagen abundance (AC) and mucoid deposition (MD) were assessed.

**Results:** Compared to N, CIH promoted significant increases in IMT (52.58 ± 2.82 μm vs. 46.07 ± 4.18 μm, *p* < 0.003), ED (25.29 ± 14.60% vs. 4.74 ± 5.37%, *p* < 0.001), EF (5.80 ± 2.04 vs. 3.06 ± 0.58, *p* < 0.001), LFF (0.65 ± 0.34% vs. 0.14 ± 0.09%, *p* < 0.001), AC (3.43 ± 1.52% vs. 1.67 ± 0.67%, *p* < 0.001) and MD (3.40 ± 2.73 μm^2^ vs. 1.09 ± 0.72 μm^2^, *p* < 0.006). ASA treatment mitigated the CIH-induced alterations in IMT: 44.07 ± 2.73 μm; ED: 10.57 ± 12.89%; EF: 4.63 ± 0.88; LFF: 0.25 ± 0.17% and AC: 0.90 ± 0.13% (p<0.05 for all comparisons).

**Conclusions:** ASA prevents the CIH-induced aortic vascular remodeling, and should therefore be prospectively evaluated as adjuvant treatment in patients with OSA.

## Introduction

Obstructive sleep apnea (OSA) is a chronic medical condition characterized by the presence of increased collapsibility of the upper airway promoting repetitive partials or complete occlusions during sleep, which lead to chronic intermittent hypoxia (CIH). This highly prevalent sleep disorder (Young et al., 2002; Fuhrman et al., 2012), has been widely associated with increased risk for cardiovascular morbidity and mortality (Drager et al., 2005; Marin et al., 2005; Campos-Rodriguez et al., 2014), along with neurocognitive and mood disorders (Li et al., 2003; Dalmases et al., 2015), metabolic dysfunction (Koren et al., 2016), as well as cancer (Almendros et al., 2012; Vilaseca et al., 2017). It is well established that OSA can trigger a group of proinflammatory and prothrombotic pathways that serve as the prelude to the initiation and propagation of atherosclerosis, the latter increasing the incidence of cardiovascular diseases (CVD) over time (Shamsuzzaman et al., 2003; Parish and Somers, 2004; Drager et al., 2011). A wealth of rodent-based studies has demonstrated that CIH, a hallmark of OSA, induces inflammation, oxidative stress, and vascular dysfunction contributing to atherogenesis (Dempsey et al., 2010; Chihara et al., 2013; Gileles-Hillel et al., 2014; Cortese et al., 2017). In addition, the increase in the sympathetic outflow elicited by hyperstimulation of the peripheral chemoreceptors in the context of CIH has been linked to higher resistance measurements in both large and small vessels, such as arterioles (Tahawi et al., 2001), which can account, at least in part for the elevation of systemic blood pressure following CIH (Fletcher et al., 1992; Bosc et al., 2010) as well as the hypertension associated to moderate/severe OSA in patients (Fletcher, 2001). Furthermore, we recently found marked aortic remodeling as illustrated by intima-media thickening and elastic fiber network disorganization, fragmentation, and alterations in the collagen content as well as mucoid deposition in the extracellular matrix in mice exposed to CIH for 6 weeks (Castro-Grattoni et al., 2016).

Several reports have proposed that over activation of cyclooxygenases (COX) may mediate the poor cardiovascular outcomes observed in OSA patients (Del Rio et al., 2012; Tual-Chalot et al., 2012; Chihara et al., 2013; Gautier-Veyret et al., 2013; Beaudin et al., 2014). The COX pathway involves cyclooxygenase-1 (COX-1) and cyclooxygenase-2 (COX-2) enzymes regulating the synthesis of several prostaglandins (PG) and a series of downstream metabolites that are functionally involved in processes such as pain, inflammation and coagulation. COX-1 is the constitutive isoform, is ubiquitously expressed in all cells and subserves homeostatic functions, while COX-2 is the inducible isoform, whose expression and activity are up-regulated in situations involving inflammation or stress (Williams et al., 1999; García and Gómez-Reino, 2000). Accordingly, inhibition of COX-1 enzyme and consequent targeting of key mediators of platelet activation and aggregation processes (Angiolillo, 2012) is commonly used in routine clinical practice for coronary and cerebral vascular disease treatment and prevention strategies, as illustrated by acetylsalicylic acid (ASA) monotherapy. In the context of OSA, the potential role of PG in the CVD morbid consequences associated with this sleep breathing disorder has been investigated (Dempsey et al., 2010; Chihara et al., 2013; Gileles-Hillel et al., 2014). However, only a few studies have tested the effects of selective or non-selective COX-1 and COX-2 inhibitors in OSA patients or CIH animal models on the expression of systemic/urine inflammatory markers (Del Rio et al., 2012; Chihara et al., 2013; Gautier-Veyret et al., 2013; Beaudin et al., 2014) and their effects on atherogenesis (Belton et al., 2003; Gautier-Veyret et al., 2013).

Therefore, we hypothesized that COX-targeted pharmacological treatment could prevent primary CVD induced by CIH. Here examined the effect of ASA, a worldwide used, safe, and cheap drug, on the previously reported aortic pro-atherogenic changes induced by CIH in a murine model of OSA (Castro-Grattoni et al., 2016). If effective, an ASA-based pharmacological intervention would be primarily expected to benefit patients at high CVD risk, such as obese OSA patients, those with co-existing morbidities, or even those patients who are non-adherent to continuous positive airway pressure therapy (CPAP). Specifically, we evaluated the preventive effect of ASA on CIH-induced aortic remodeling by assessing relevant histopathological features, namely collagen accumulation, elastin fiber disorganization/fragmentation, intima-media thickness and mucoid deposition in the aortic wall, in mice exposed to CIH or normoxia for a 6-week period while being treated with either ASA or placebo.

## Methods

The study was performed on 40 pathogen-free (C57BL/6, 6-week-old, male) mice (Charles River Laboratories, Saint Germain sur L'arbresle, France). The animals were maintained on a 12-h light/dark cycle, and fed with a regular diet chow and tap water *ad libitum*. All experimental procedures were approved by the Ethical Committee for Animal Research at the University of Barcelona. Mice were randomly assigned into 4 groups (10 mice per group): (1) CIH exposure + ASA (CIH+A); (2) CIH exposure + placebo (CIH) or (3) normoxia + ASA (N+A) and (4) normoxia + placebo (N). Intermittent hypoxia was applied for 6 h·day^−1^ during the light period (10:00–16:00 h) for a total of 6 weeks.

The experimental setting has been previously described (Almendros et al., 2012), and was used to subject mice to CIH at a rate of 60 events·h^−1^, each event consisting of 20 s of 5% O_2_ followed by 40 s of room air. Briefly, a continuous flow of gas was circulated through a box (26 cm long, 18 cm wide and 6 cm high) by means of a distribution system based on multiple orifices. A pneumatic valve placed near the inlet of the box cyclically switched from a room air entrance (40 s) to a gas reservoir of hypoxic air at an oxygen fraction of 5% (20 s) for CIH, or room air for normoxia. At the end of the experiment, the mice were anesthetized (urethane 20%, 1 g/kg) and euthanized by exsanguination. Then, the mid-thoracic aorta was excised, perfused with phosphate-buffered saline, fixed with 4% paraformaldehyde and paraffin-embedded for further histological analysis.

In the groups exposed to CIH and N and treated with ASA, mice were administered a daily dose of 75 mg/kg body-weight. To this end, a granulated formulation of ASA (Aspirin Bayer ® 500 mg) was dissolved into their drinking water obtaining a concentration of 500 mg/L. Considering that each animal drinks of 3 mL of water per day on average, this would correspond to 1.5 mg ASA per day for a mouse of 20 g of weight. This dose was selected based on previous reports showing this dosage's ability to suppress platelet COX in mice (Praticò et al., 2000; Armstrong et al., 2011). The animals in the CIH and N groups with placebo received tap water.

The samples obtained from paraffin blocks were stained with hematoxylin and eosin (H&E, Master Diagnostica, Granada, Spain), Gomori trichrome stain (Artisan Link Special Staining System; DAKO, Santa Clara, California, United States) or Alcian blue (Alcian blue 2.5; Bio-Optica, Milano, Italy). Furthermore, the images obtained for measurement analysis (from four consecutive sections per sample) were processed using Image J (National Institutes of Health) and Adobe Photoshop CS6 (Adobe Systems Inc) software. For image capture, a digital microimaging network instrument (Leica-DMD-108; Leica Microsystems) was used, and aortic auto-fluorescence was visualized using a fluorescence microscope (Olympus-BX51; Olympus) (Castro-Grattoni et al., 2016). The intima media thickness (IMT) was quantified from H&E preparations (300 measurements from each animal). Elastic fiber analysis was performed using aortic auto-fluorescence to quantify: elastic fragmentation (EF) (i.e., the complete fragmentation of one elastic fiber), length between fragmented fiber endpoints (LFF) (adjusted by total aortic area and shown as percent space without fiber) and elastin disorganization (ED). Finally, for mucoid deposition (MD) and aortic wall collagen (AC), the integrated density of the Alcian blue and Gomori trichrome stains respectively, were quantified and adjusted to the corresponding aortic area (Castro-Grattoni et al., 2016). An investigator blinded to the experimental group performed all of the analyses.

Data are reported as mean ± standard deviation. Shapiro-Wilk tests were performed to ensure normal distribution of samples prior to analysis. Data from N, N+A, CIH, and CIH+A groups were compared using Two-Way Analysis of Variance. CIH *vs*. N exposures and ASA administration *vs*. placebo constituted the variables. Student-Newman-Keuls post hoc test was used for multiple comparisons. A p value less than 0.05 was considered as the cut-off for statistical significance.

## Results

At baseline, body weights were similar among all groups. After 6 weeks of CIH, mice experienced significant weight loss, corresponding to ~10.8% from baseline (*p* < 0.001). However, ASA treatment did not have any effect on animal body weight in normoxia or CIH.

### Elastin disorganization, elastin fragmentation and length between fragmented fiber end-points

**Figure 1:**
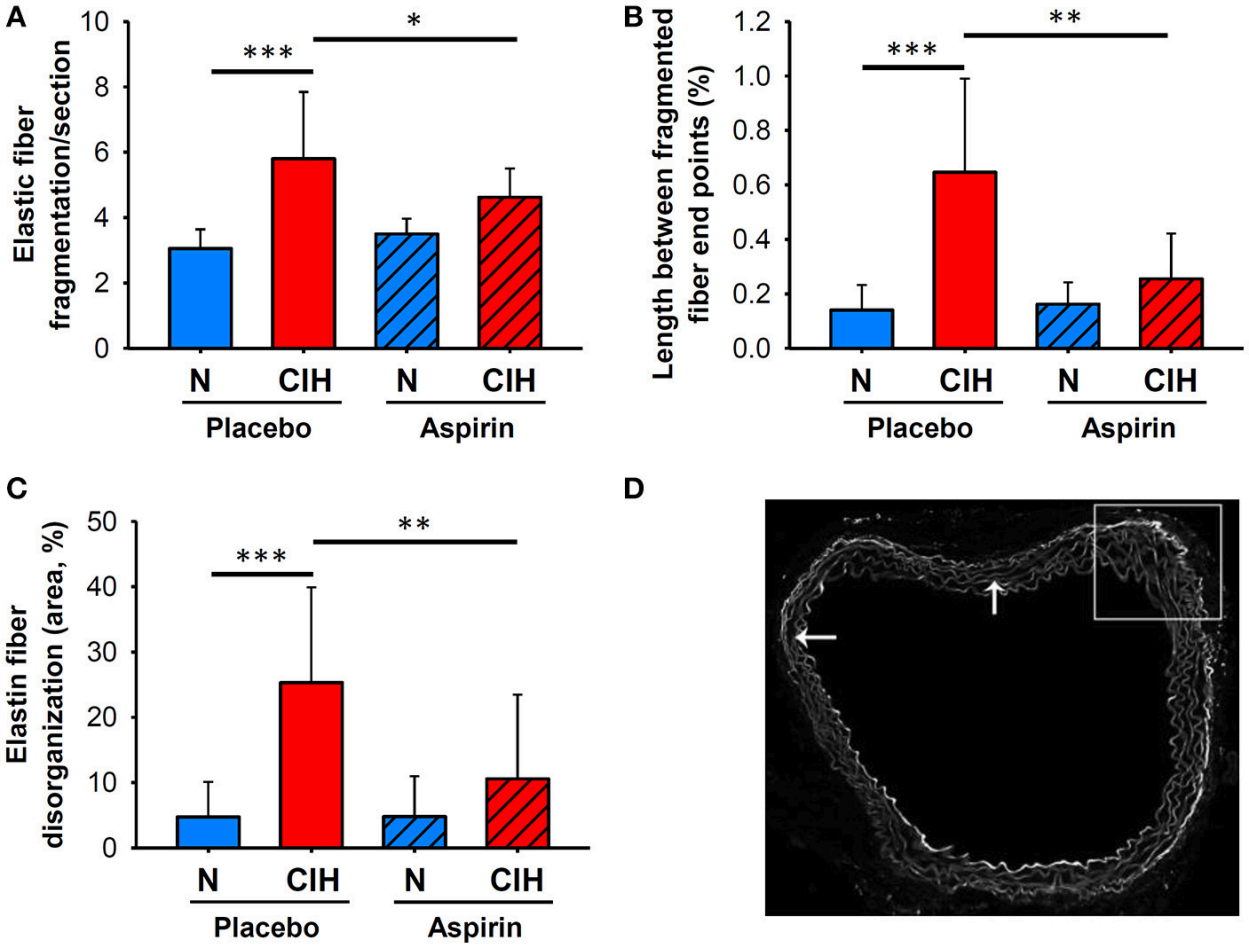
Elastin network morphological remodeling associated with CIH and ASA treatment. **(A)** Quantification of EF per section, and LFF adjusted by total aortic wall area **(B)** (shown as %). **(C)** ED area in the aortic wall adjusted by total area (shown as %). **(D)** Representative images of the elastic network (scale bar = 50 μm), revealed by auto-fluorescence, with arrows indicating fragmented elastic fiber end points. (*p < 0.05; **p < 0.01; ***p < 0.001).

The aortas of mice exposed to CIH showed increased ED (25.29 ± 14.60%) compared to those under normoxia (4.74 ± 5.37%, *p* < 0.001). Moreover, a marked increase of EF (number per section) index emerged in mice exposed to CIH (5.80 ± 2.04) in comparison with normoxia (3.06 ± 0.58, *p* < 0.001). Similarly, an increase of LFF was observed in mice exposed to CIH (0.65 ± 0.34 %) versus normoxia (0.14 ± 0.09 %, *p* < 0.001). All these histological alterations were significantly attenuated in the CIH+A group when compared with CIH (Figure 1), such that ASA treatment prevented the CIH-induced alterations, as follows: ED 10.57 ± 12.89 %, *p* < 0.007; EF 4.63 ± 0.88, *p* = 0.047 and the LFF 0.25 ± 0.17 %, *p* < 0.003 (Figure 1).

### Aortic fibrosis

**Figure 2:**
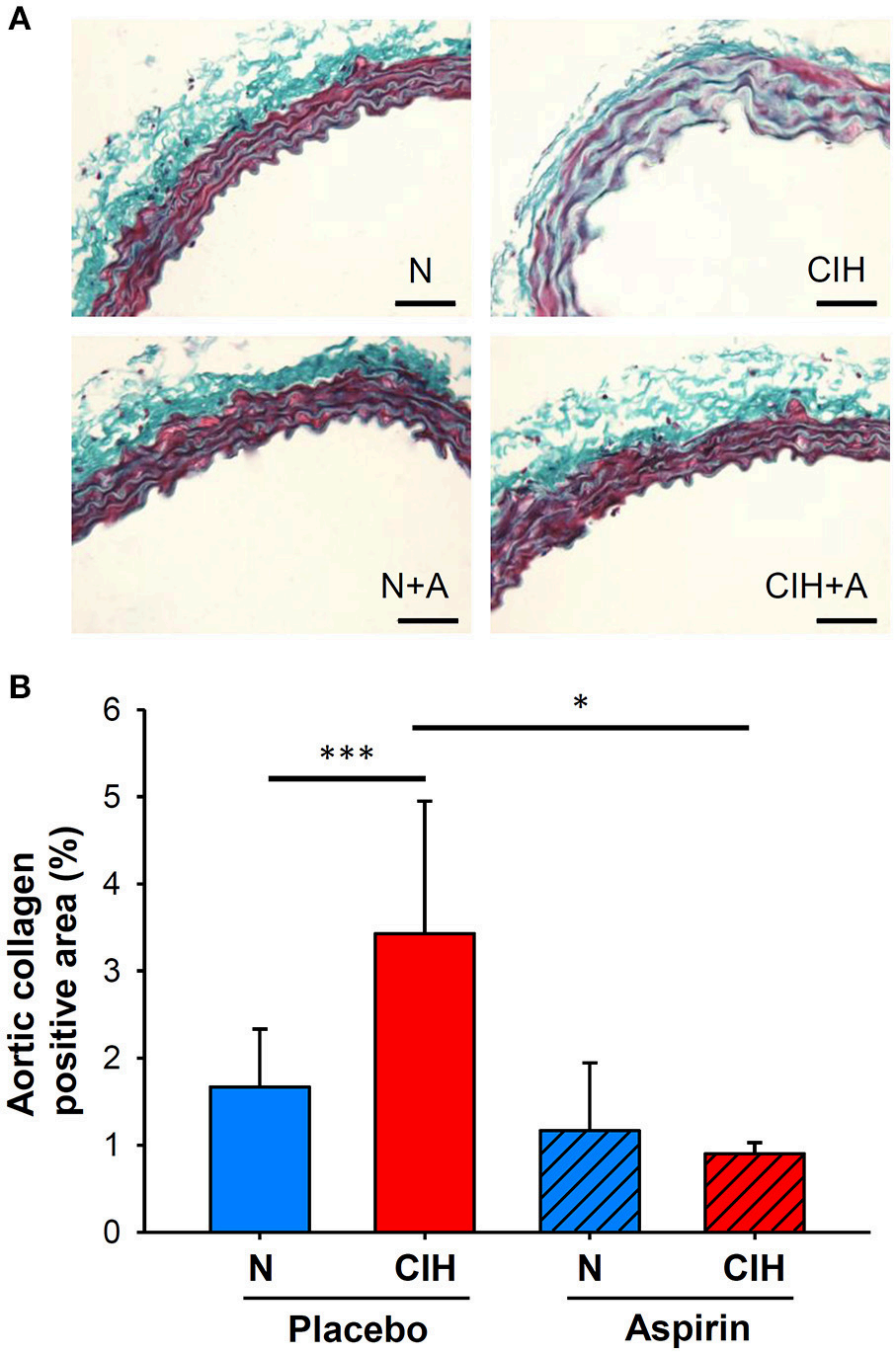
**(A)** Representative images of Gomori trichrome stain to measure aortic wall fibrosis (scale bar = 50 μm; collagen in green). **(B)** Collagen-positive area of the intima-media (%). (*p < 0.05; ***p < 0.001).

A pro-fibrotic process in the aortic wall was induced after 6 weeks of CIH exposure, as indicated by the increased AC positive area (3.43 ± 1.52%) in comparison with normoxia (1.67 ± 0.67%, *p* < 0.001). ASA treatment prevented the increases in AC positive area of the aortic wall induced by CIH (0.90 ± 0.13%, *p* = 0.034) (Figure 2).

### Intima-media thickness

**Figure 3:**
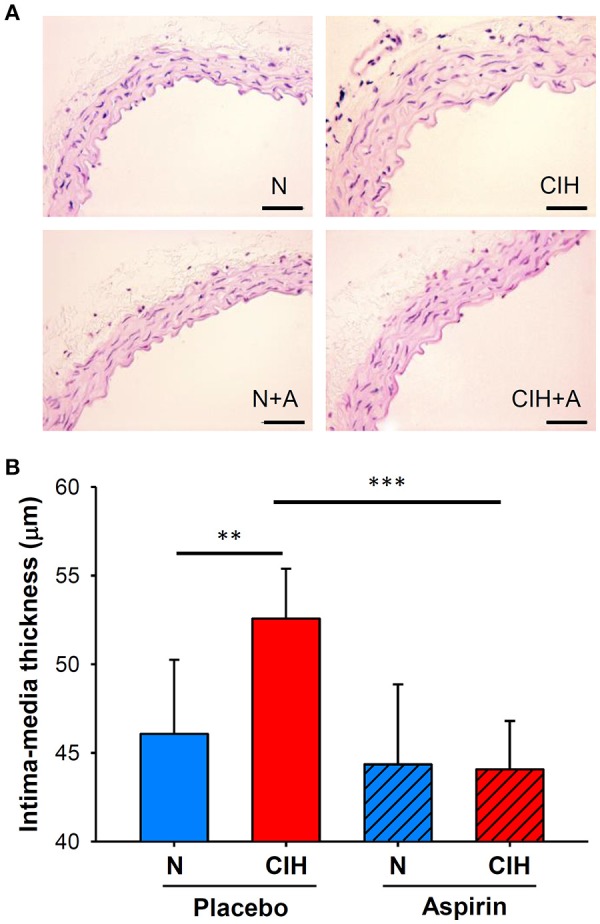
**(A)** Representative images of the aortic wall with H&E staining for each group (scale bar = 50 μm). **(B)** Histomorphometric analysis of IMT (shown as μm), H&E: hematoxylin and eosin. (**p < 0.01; ***p < 0.001).

The application of CIH promoted increases in IMT (52.58 ± 2.82 μm) when compared to mice under normoxic conditions (46.07 ± 4.18 μm; *p* < 0.003). IMT alterations induced by CIH were prevented by ASA treatment (44.07 ± 2.73 μm; *p* < 0.001; Figure 3).

### Mucoid deposition

**Figure 4:**
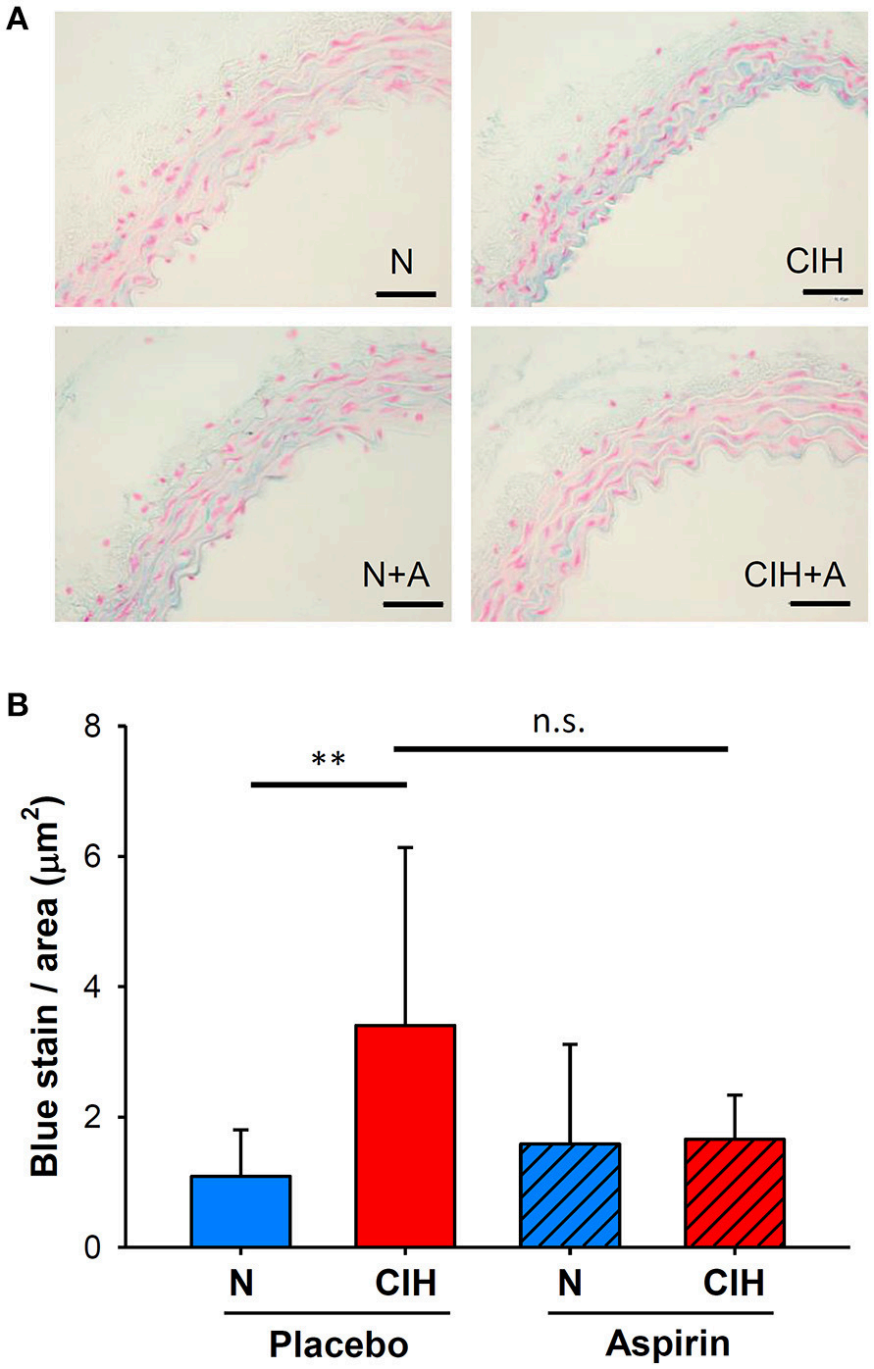
Aortic extracellular matrix remodeling induced by CIH and ASA treatment. **(A)** Representative images of Alcian blue staining from the mid thoracic aorta to detect aortic wall MD (scale bar = 50 μm; mucins in blue). **(B)** Intima-media MD shown as the ratio of the total blue density to the total aortic wall area (μm^2^). (**p < 0.01; n.s = non-significant).

The histological quantification of the positive blue area in the aortic intima-media following Alcian Blue staining revealed increases in MD within the aortic interlaminar space of mice exposed to CIH (3.40 ± 2.73 μm^2^) in comparison with normoxia (1.09 ± 0.72 μm^2^, *p* < 0.006). However, ASA treatment under CIH conditions did not significantly alter MD (1.66 ± 0.68 μm^2^, *p* = 0.188; Figure 4).

## Discussion

**Figure 5:**
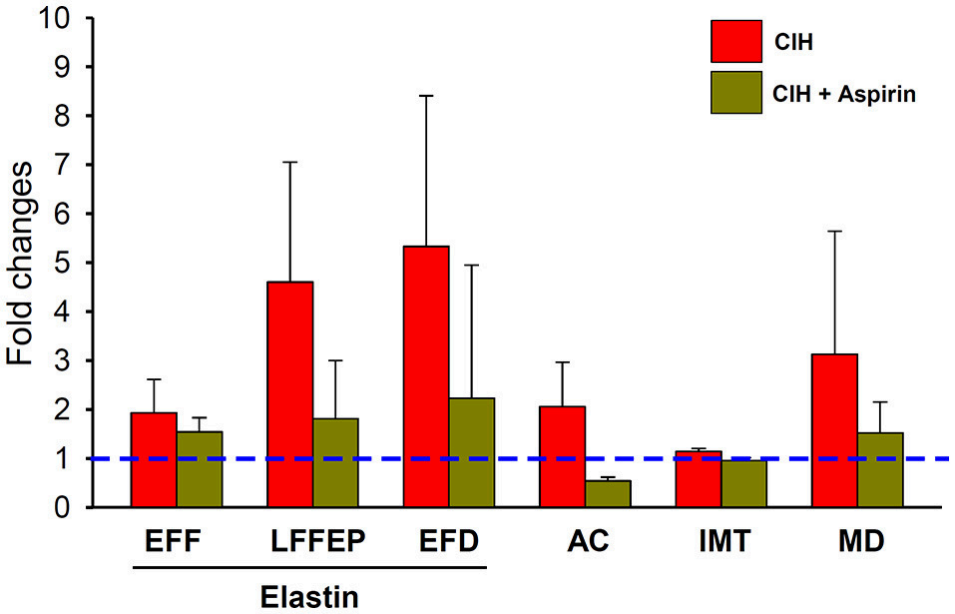
Changes induced on histopathological measurements (EF, LFF, ED, AC, IMT, and MD) in response to CIH (red bar) and CIH+ASA (yellow bar) normalized to the normoxia group. Dashed blue line represents the normoxia+placebo reference normalized value). All CIH-induced changes (except in MD) were significantly prevented by ASA treatment.

This study in a mouse model of OSA shows, for the first time, that ASA treatment concurrent with CIH exposures attenuated the development of well-established early features of atherosclerosis (as summarized by Figure 5). Therefore, the present findings strongly suggest that the COX pathway plays an important role in the vascular consequences of OSA, and most importantly, that the administration of a non-selective COX inhibitor such as ASA may help prevent the damage on the vascular wall induced by CIH during the rest period.

The repetitive episodes of hypoxia and re-oxygenation in OSA patients can occur more than 60 times per hour (Song et al., 2015), and the experimental model employed here reliably reproduced these conditions. After 6 weeks of CIH, the histopathological analysis of the aortas revealed elastin fiber and collagen remodeling, with increased IMT and MD in mice, as previously described (Castro-Grattoni et al., 2016) and in accordance with other reports (Dematteis et al., 2008; Xu et al., 2015). These results are also concordant with data from clinical studies describing that in patients with OSA, IMT is increased in the common carotid artery (Minoguchi et al., 2005; Hui et al., 2012; Gunnarsson et al., 2014), a finding that is considered as an independent risk factor for CVD (Pignoli et al., 1986; O'Leary et al., 1999; Touboul et al., 2007). In addition to IMT increases in CIH, histomorphological changes of the elastin network organization around the vascular smooth muscle cells in the aortic wall have been described in response to CIH (Chatzizisis et al., 2007). Alterations in the continuity of the elastic network with disruption of the elastic fiber arrangements have been previously implicated in the early phases of atherosclerosis in pathogen-free Sprague-Dawley rats (Xu et al., 2015) as well as in mice (Castro-Grattoni et al., 2016) exposed to CIH. As illustrated by current findings, the elastic fiber staining showed an irregular pattern of elastic fiber distribution after CIH. Furthermore, the accumulation of extracellular matrix proteins, particularly collagen and fibronectin in the vascular media has also been reported as a characteristic feature indicative of the development of atherosclerosis (Lan et al., 2013). In this regard, the exposure to CIH can also trigger an increased collagen deposition in the tunica media (Philippi et al., 2010), consistent with our results. However, contrary to the present study no differences on IMT were found after CIH (Lan et al., 2013). The deposition of mucoid material within the intima and media of the arterial wall has also been described, and assumed to reflect a precipitating factor underlying vascular wall weakening and increased risk for aneurysm formation (Katz et al., 2008). Indeed, Arnaud et al. evaluated aorta remodeling caused by CIH and showed enhanced mucoid accumulation between sub-intimal elastic fibers, which could result from elastin degeneration or mucoid degenerating vascular smooth muscle cells (Arnaud et al., 2011).

There is increasing evidence that chronic inflammation and oxidative stress participate in the increased risk of cardiovascular pathophysiology in the context of OSA. This notion has been mainly supported by *in vitro* and *in vivo* studies describing the activation of inflammatory pathways in response to CIH (Nanduri and Nanduri, 2007; Dematteis et al., 2008; Lavie and Polotsky, 2009; Lévy et al., 2009; Ryan and McNicholas, 2009; Arnaud et al., 2011; Tuleta et al., 2015; Xu et al., 2015; Chen et al., 2016; Liu et al., 2018). Furthermore, we have recently shown that the inflammatory effects of CIH exposures during sleep lead to the recruitment of activated macrophages to the aortic wall initiating and propagating the atherogenesis cascade (Cortese et al., 2017). In addition, CIH enhances peripheral chemoreceptor reflexes, fostering enhanced tonic and reactive sympathetic outflow responses that foster increased vascular resistance, thereby contributing to the hypertension associated with OSA (Fletcher, 2001). Taken together, these inflammatory and vasoactive effects of CIH could, if left untreated, promote the emergence of adverse cardiovascular events, as manifested by early hemodynamic alterations along with histologic vascular remodeling (Dematteis et al., 2008; Castro-Grattoni et al., 2016). Among these pathways, there are some laboratories that have pointed to the development of cardiovascular injury in the context of increased activation of the COX pathway (Jelic et al., 2008; Lévy et al., 2009; Ryan et al., 2009; Tuleta et al., 2015; Micova et al., 2016). In a recent study by Gautier-Veyret and collaborators, COX-1 was activated in mice exposed to CIH, and treatment with a specific COX-1 inhibitor reduced the magnitude of CIH-induced lesions in the aorta (Gautier-Veyret et al., 2013). Increased levels of COX metabolites have been consistently reported in response to cyclic hypoxia by several cell types including endothelial cells (Li et al., 2003; Daneau et al., 2010; Tang et al., 2014), vascular smooth muscle cells (Tang et al., 2014) and immune cells (Campillo et al., 2017). Furthermore, clinical studies have associated COX-activity with common carotid artery intima media thickness (CCA-IMT). Wohlin et al. performed a backward-stepwise regression analysis, and found that PG-F2α was independently associated with CCA-IMT in their cohort (Wohlin et al., 2007). Similarly, Beloqui and colleagues found significant associations between PG-E2 and CCA-IMT, after adjustments for cardiovascular and inflammatory risk factors (Beloqui et al., 2005). In this regard, the murine studies examining the COX pathway in the context of CIH have undoubtedly provided compelling evidence on the putative role of COX in CIH-induced vascular remodeling. However, these studies used pharmacological agents targeting COX that are seldom used in clinical practice. Here, we purposefully opted instead for the administration of the commonly used COX inhibitor, ASA, in light of its well established beneficial role in the prevention of cardiovascular outcomes (Kwok and Loke, 2010). Although ASA is typically viewed as a non-specific COX inhibitor, it is highly selective for COX-1 vs. COX-2 (Ornelas et al., 2017). ASA treatment effectively prevented CIH-induced aortic remodeling changes on nearly all the measures that were evaluated. However, we should also note that this work has some limitations that will need to be explored in further studies: (i) Chronic ASA treatment could also be beneficial and attenuate the hypertension induced by CIH (Fletcher et al., 1992; Bosc et al., 2010), as recently reported in hypertensive rats (Maji et al., 2018). The occurrence of such sustained elevation of systemic blood pressure could be also an important contributor to the aortic remodeling induced by CIH. In addition, a recent study showed the divergent roles of COX-1 and COX-2 in modulating vascular responses to IH, whereby human subject exposed to acute IH manifested increases in arterial blood pressure, which were abrogated by selective COX-2 inhibition (Beaudin et al., 2014). However, we should remark that (ii) treatment with ASA can result in an irreversible acetylation of the COX promoter resulting in a quasi-permanent inhibition of platelet function. Indeed, the inactivation of COX-1 by ASA leads to long-lasting suppression of thromboxane (TX) A2 production and TXA2-mediated platelet activation and aggregation (Patrono, 2015). In addition, the production of other prostanoids by other cell types could be also modulated by cell-specific ASA responses, pharmacokinetics and ASA differential selectivity for COX-1 vs. COX-2 (Warner et al., 2011).

Considering the lasting and sustained CVD-related benefits derived from routine low-dose ASA treatment, treatment options that limit the activity of the COX pathway in patients with OSA could be highly relevant for the prevention and treatment of their enhanced CVD risk. Of note, the activity of the COX pathway, production of PG, their metabolites and the efficacy of COX inhibitors have yet to be comprehensively evaluated in patients with OSA. Considering that more than 30% of patients with OSA either do not tolerate CPAP treatment or become non-adherent (Weaver and Grunstein, 2008; Wozniak et al., 2014), failure to implement any alternative intervention would maintain the elevated risk of CVD in these patients. We propose that in light of the safety profile and extensive experience with low dose ASA in clinical settings, addition of ASA to the therapeutic regimen of all OSA patients, and particularly to those at high risk of CVD morbidity or those not receiving CPAP should be contemplated, critically evaluated, and possibly implemented.

In conclusion, the present study reveals the positive and dampening effect of a common non-selective COX inhibitor such as ASA on the aortic remodeling induced by CIH in mice. These findings do not only corroborate the role of COX-related pathways in the pathophysiology of vascular disease in OSA, but also point to the potential benefits of pharmacological treatment targeting this pathway in patients with OSA, particularly in those at high risk for cardiovascular disease.

## Author contributions

IA, CP, MS-G, and RF participated in the conceptual framework of the project. MS-G, AC-G, and MT performed experiments and analyzed the experimental data. IA, MS-G, CP, RF, MS-d-l-T, FB, DG, and JM interpreted the results. MS-G and IA participated in the drafting of the manuscript, which was reviewed, edited, and approved by all authors. IA, RF, and JM are responsible for the financial support of the project. IA is the guarantor of the paper.

### Conflict of interest statement

The authors declare that the research was conducted in the absence of any commercial or financial relationships that could be construed as a potential conflict of interest.

## Abbreviations

AC: aortic wall collagen
ASA: acetylsalicylic acid
CCA-IMT: common carotid artery intima media thickness
CIH: chronic intermittent hypoxia, chronic intermittent hypoxia plus placebo
CIH+A: chronic intermittent hypoxia exposure plus acetylsalicylic acid
COX: cyclooxygenase
COX-1: cyclooxygenase 1
COX-2: cyclooxygenase 2
CPAP: continuous positive airway pressure therapy
CVD: cardiovascular diseases
ED: elastin disorganization
EF: elastin fragmentation
H&E: hematoxylin and eosin
IMT: intima-media thickness
LFF: length between fragmented fiber endpoints
MD: mucoid deposition
N+A: normoxia plus acetylsalicylic acid
N: normoxia plus placebo, Normoxia
OSA: obstructive sleep apnea
PG: prostaglandins.
